# Role of Tyrosine Nitrosylation in Stress-Induced Major Depressive Disorder: Mechanisms and Implications

**DOI:** 10.3390/ijms241914626

**Published:** 2023-09-27

**Authors:** Gregory C. Wilson, Simone Keitsch, Matthias Soddemann, Barbara Wilker, Michael J. Edwards, Erich Gulbins

**Affiliations:** 1 Department of Surgery, University of Cincinnati College of Medicine, Cincinnati, OH 45267, USA; wilsong3@ucmail.uc.edu; 2 Institute of Molecular Biology, University Hospital Essen, University of Duisburg-Essen, Hufelandstrasse 55, 45122 Essen, Germany; simone.keitsch@uk-essen.de (S.K.); matthias.soddemann@uk-essen.de (M.S.); barbara.wilker@uk-essen.de (B.W.); turninflat@icloud.com (M.J.E.)

**Keywords:** major depressive disorder, tyrosine nitrosylation, antidepressants, ceramide, phosphatidic acid, neuroinflammation, neuroplasticity, hippocampus, peroxidases, catalase

## Abstract

Major depressive disorder (MDD) has a lifetime prevalence of approximately 10% and is one of the most common diseases worldwide. Although many pathogenetic mechanisms of MDD have been proposed, molecular details and a unifying hypothesis of the pathogenesis of MDD remain to be defined. Here, we investigated whether tyrosine nitrosylation, which is caused by reaction of the C-atom 3 of the tyrosine phenol ring with peroxynitrate (ONOO^−^), plays a role in experimental MDD, because tyrosine nitrosylation may affect many cell functions altered in MDD. To this end, we induced stress through glucocorticoid application or chronic environmental unpredictable stress and determined tyrosine nitrosylation in the hippocampus through immuno-staining and ELISA. The role of catalases and peroxidases for tyrosine nitrosylation was measured using enzyme assays. We show that glucocorticoid- and chronic unpredictable environmental stress induced tyrosine nitrosylation in the hippocampus. Long-term treatment of stressed mice with the classical antidepressants amitriptyline or fluoxetine prevented tyrosine nitrosylation. Tyrosine nitrosylation was also prevented through i.v. application of anti-ceramide antibodies or recombinant ceramidase to neutralize or degrade, respectively, blood plasma ceramide that has been recently shown to induce experimental MDD. Finally, the application of phosphatidic acid, previously shown to be reduced in the hippocampus upon stress, also reverted stress-induced tyrosine nitrosylation. The inhibition of tyrosine nitrosylation by interfering with the formation of NO radicals at least partly restored normal behavior in stressed mice. These data suggest that tyrosine nitrosylation might contribute to the pathogenesis of MDD and targeting this process might contribute to the treatment of MDD.

## 1. Introduction

Major depressive disorder (MDD) is one of the most common diseases with a lifetime prevalence of approximately 10% [1]. Patients with MDD often suffer from severely depressed mood, the inability to feel, loss of interest, anhedonia, fear, feelings of worthlessness, weight loss, insomnia, and concentration deficits, but also have increased cardiovascular disease, increased levels of inflammatory cytokines, osteoporosis, and adrenocortical activation [1,2,3,4,5,6,7]. The combination of these findings suggests that MDD is a systemic disease and not localized to the central nervous system. Severe MDD has a relatively high mortality of approximately 10% from suicide. Further, approximately 50% of all patients with MDD think about suicide and approximately 30% undergo suicide attempts [8]. MDD accounts for approximately 85% of all completed suicides, supporting the importance of treating MDD [8].

A variety of pathogenetic mechanisms of MDD have been suggested, but the pathogenesis of MDD is still unknown and the molecular mechanisms causing MDD still need to be elucidated. It has been shown that the concentration, distribution, and function of neurotransmitters, particularly monoamines, are altered in MDD. However, the monoamine hypothesis of major depression has been questioned because some antidepressants, such as tianeptine, do not prevent but rather enhance serotonin reuptake [9] or do not act via the serotonin system at all, such as ketamine, which targets N-methyl-D-aspartate (NMDA) receptors [10,11,12]. Other hypotheses to explain the pathogenesis of MDD include alterations of synapses in the hippocampus and frontal brain [13]; however, it remains unknown whether a decrease in synapsis density is a cause or consequence of MDD. Other studies focused on cytokines and stress hormones in MDD and showed diminished concentrations of growth factors such as BDNF [14], systemic changes of the glucocorticoid system, and alterations of cytokine concentrations such as Interleukin-6 in the brain [15]. In particular, cytokines such as IL-6 are able to induce symptoms of MDD, but it remains unknown whether an increase in IL-6 is a true pathogenetic factor in MDD. Additional studies demonstrated dysfunctions of endothelial cells and the blood–brain barrier [16,17,18,19], dysfunctions of receptors causing aberrant neuroplasticity [20,21], a reduced proliferation of hippocampus neurons [22,23,24], and a dysregulation of autophagy [25] as pathogenetic factors of MDD. The studies on endothelial cells in stressed mice that demonstrated a down-regulation of claudin 5 expression in endothelial cells in the nucleus accumbens upon stress, resulting in blood–brain-barrier leakiness [16], provide a potential mechanism to how increased blood levels of Interleukin-6 may contribute to the pathogenesis and the symptoms of MDD. This notion is supported by findings that the permeability of the blood–brain barrier was increased in a mouse model of chronic-stress-induced MDD, which was mediated by changes in the VEGF/VEGFR2 system [19]. Although classical antidepressants correct the reduced neuronal proliferation in MDD over a time period of 2–3 weeks [22,23,24], the hypothesis that reduced neurogenesis causes MDD fails to explain why irradiation-induced inhibition of neuronal proliferation does not necessarily induce MDD and why drugs such as ketamine or electroconvulsive therapy act much faster against MDD.

Obviously, the etiology of MDD is likely multifactorial and requires definition. It is also necessary to identify mechanisms that could connect the different findings on the pathogenesis of MDD and provide a potential common molecular mechanism resulting in many of the above-described findings such as reduced proliferation, the reduced formation of synapses, reduced concentrations of growth factors, or alterations in the endothelial cell-barrier.

Several recent studies have shown that ceramide is increased in the blood plasma of human individuals with MDD, which is paralleled by an increase in ceramide in mice upon application of several forms of stress [26,27,28,29,30]. The increase in ceramide in the blood plasma of patients with MDD was independently shown by four groups [26,27,28,29,30]. The significance of the increase in blood plasma ceramide in MDD was studied by our group by investigating the effect of intravenous injection of anti-ceramide antibodies that bind and neutralize ceramide or neutral ceramidase that cleaves ceramide [26]. These treatments rapidly, i.e., within 24 h, abrogated stress-induced MDD [26]. Conversely, intravenous injection of blood plasma that was incubated and loaded ex vivo with ceramide induced MDD in previously untreated mice [26]. These studies further demonstrated that ceramide inhibited the activity of phospholipase D (PLD) in hippocampal endothelial cells, which resulted in reduced concentrations of phosphatidic acid, the product of PLD activity, in the hippocampus of stressed mice [26]. Treatment of depressed mice with intravenous phosphatidic acid rapidly reverted MDD, indicating the significance of phosphatidic acid for MDD [26]. Ceramide release upon stress was mediated by the neutral sphingomyelinase 2 [30]. We further demonstrated that phosphatidic acid binds to and inhibits protein tyrosine phosphatase 1B (PTP1B) in the hippocampus, thereby promoting cellular tyrosine phosphorylation, which counteracted MDD [31].

Here, our aim was to identify common targets of phosphatidic acid and classical antidepressants in an effort to define important pathways for the molecular mechanisms of action for different antidepressants, but also to define the molecular pathogenesis of MDD.

To this end, we investigated whether stress induces tyrosine nitrosylation and the effects of antidepressive treatments on tyrosine nitrosylation. Tyrosine nitrosylation is mediated at C-atom 3 of the phenol ring through the reaction with peroxynitrate (ONOO^−^), which is formed through the reaction of NO with O_2_^−^-radicals, or through reaction with NO_2_ [32]. Tyrosine nitrosylation may interfere with the function of many proteins, and since tyrosine nitrosylation might be irreversible, repair may require a novel synthesis of the affected proteins. Thus, tyrosine nitrosylation is an attractive target in MDD, because tyrosine nitrosylation may involve several signaling pathways and functions in neurons and could explain the variety of mechanisms involved in MDD described above.

We aimed to investigate whether different forms of stress induce tyrosine nitrosylation in the hippocampus and whether this event is prevented by classical antidepressants such as amitriptyline or fluoxetine, but also by the neutralization or degradation of blood plasma ceramide or by application of phosphatidic acid. We also tested whether the inhibition of tyrosine nitrosylation by interfering with the formation of NO radicals impacts the behavior of stressed mice.

## 2. Results

### 2.1. Stress Induces Tyrosine Nitrosylation in the Hippocampus

**Figure 1:**
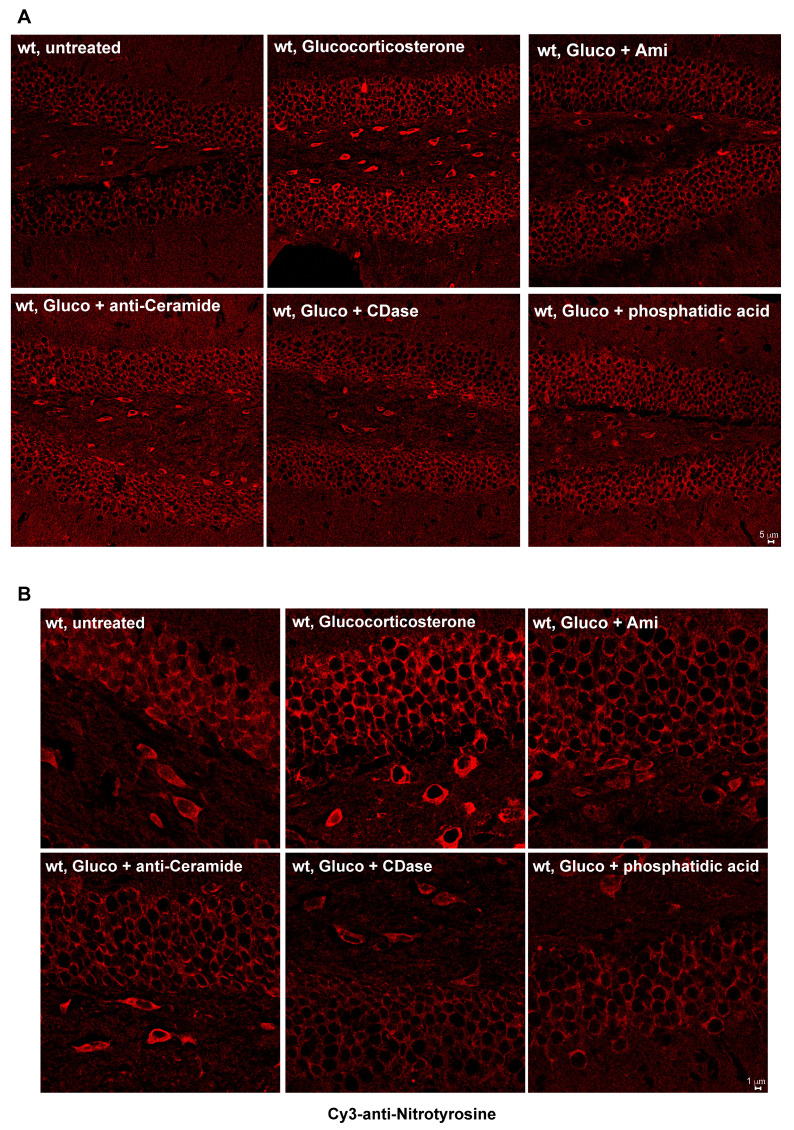

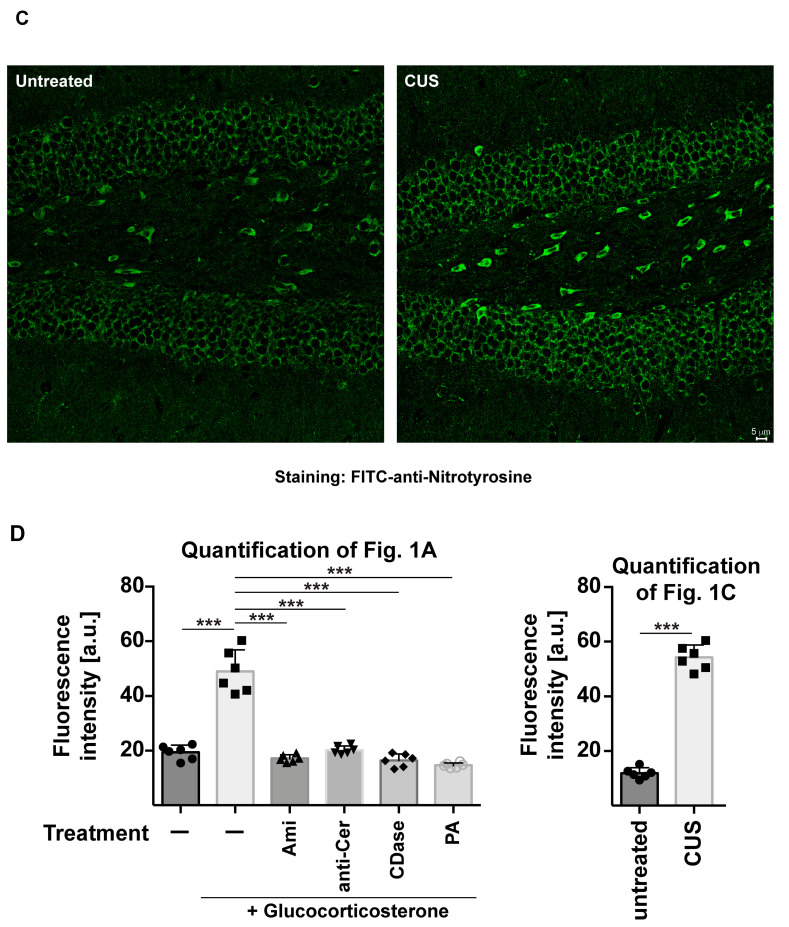
Stress induces nitrosylation in the hippocampus, which is prevented by treatment with antidepressants, anti-ceramide antibodies, recombinant ceramidase, or phosphatidic acid. Tyrosine nitrosylation in the hippocampus was determined by staining of hippocampus sections with anti-nitrotyrosine antibodies. (**A**,**B**) Glucocorticoid stress induces tyrosine nitrosylation in the hippocampus, which, in the glucocorticosterone-stressed mice, is prevented through treatment with amitriptyline (Ami), anti-ceramide antibodies, recombinant acid ceramidase, or phosphatidic acid. Panel (**B**) shows increased magnification of the hippocampus. (**C**) Tyrosine nitrosylation is also induced by chronic unpredictable stress (CUS). Shown are representative confocal microscopies from 6 independent studies and the quantification of the fluorescence (**D**) giving the mean ± SD from 6 independent samples (mice). On each slide, we quantified fluorescence in 20 neurons and determined the mean. These means are given as values. *** *p* < 0.001, ANOVA with Tukey’s post hoc test.

To determine whether major depression induced by glucocorticoid stress or chronic unpredictable stress triggers the nitrosylation of proteins in the hippocampus, we stained sections from unstressed (resting), stressed, or stressed and treated mice with anti-nitrotyrosine antibodies. Treatment consisted of a 14-day treatment with amitriptyline or fluoxetine via drinking water or of two i.v. injections of anti-ceramide antibodies, recombinant ceramidase or phosphatidic acid, 24 and 12 h prior to preparation of the brains. The results indicate that both glucocorticoid stress and chronic unpredictable stress triggered tyrosine nitrosylation in the hippocampus, which was prevented through treatment with amitriptyline and fluoxetine, as well as injections of anti-ceramide antibodies, recombinant ceramidase, or phosphatidic acid (Figure 1A–D).

**Figure 2:**
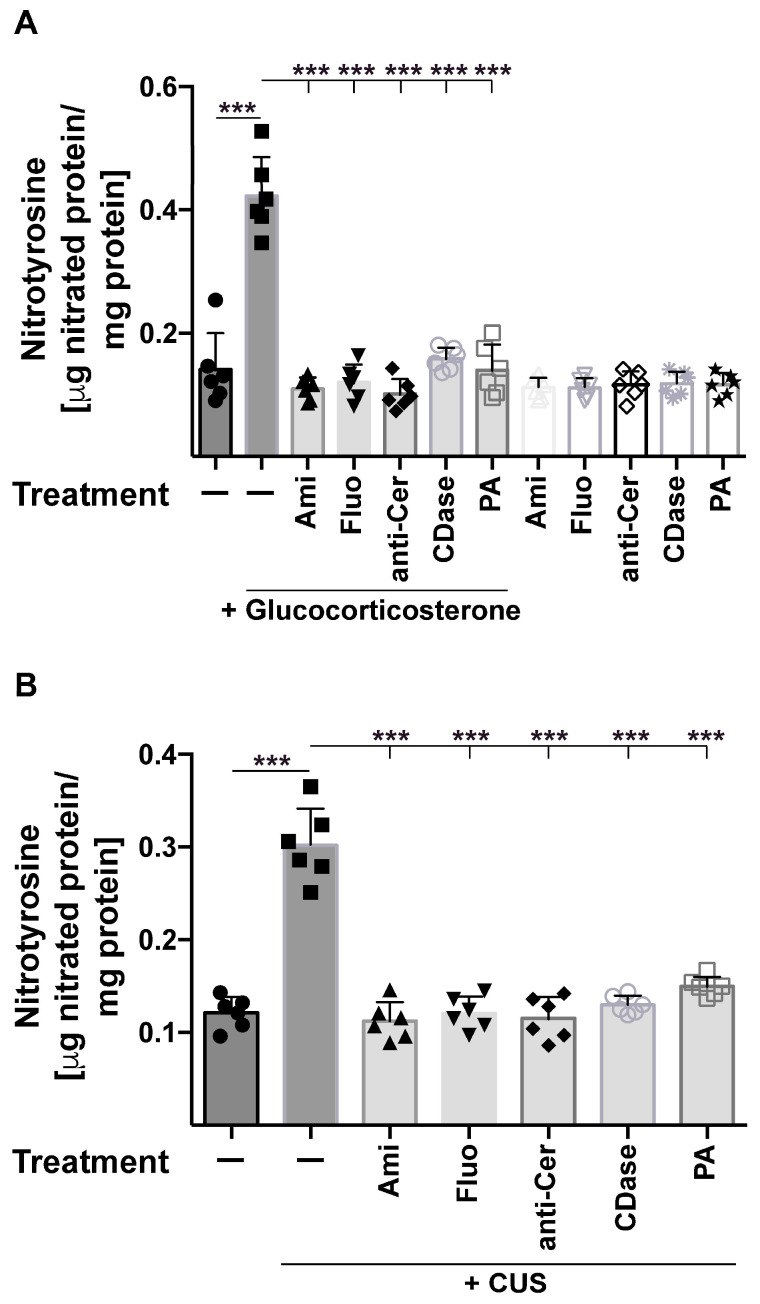
Quantitative analysis of nitrotyrosine residues in the hippocampus after stress and treatment of the mice with antidepressants, anti-ceramide antibodies, recombinant ceramidase, or phosphatidic acid. Tyrosine nitrosylation in the hippocampus was determined through quantitative ELISA for nitrotyrosine residues upon application of glucocorticoid stress (**A**) or chronic unpredictable stress (CUS) (**B**). Mice were treated with amitriptyline (Ami), fluoxetine (Fluo), anti-ceramide antibodies (anti-Cer), recombinant ceramidase (CDase), or phosphatidic acid (PA) or were left untreated (-). Shown are the mean ± SD from 6 independent studies; statistical differences were determined using ANOVA with Tukey’s post hoc test; *** *p* < 0.001.

Next, we quantified nitrated proteins in the brain using an ELISA assay to detect nitrotyrosine residues in hippocampus homogenates. The studies revealed a marked increase in nitrotyrosine residues after glucocorticoid stress or chronic unpredictable stress (Figure 2A,B). Nitrosylation was again prevented by treatment of the mice with amitriptyline and fluoxetine, as well as injections of anti-ceramide antibodies, recombinant ceramidase, or phosphatidic acid (Figure 2A,B).

### 2.2. Stress-Induced Tyrosine Nitrosylation Is Not Mediated by an Increased Activity of NO-Synthases

**Figure 3:**
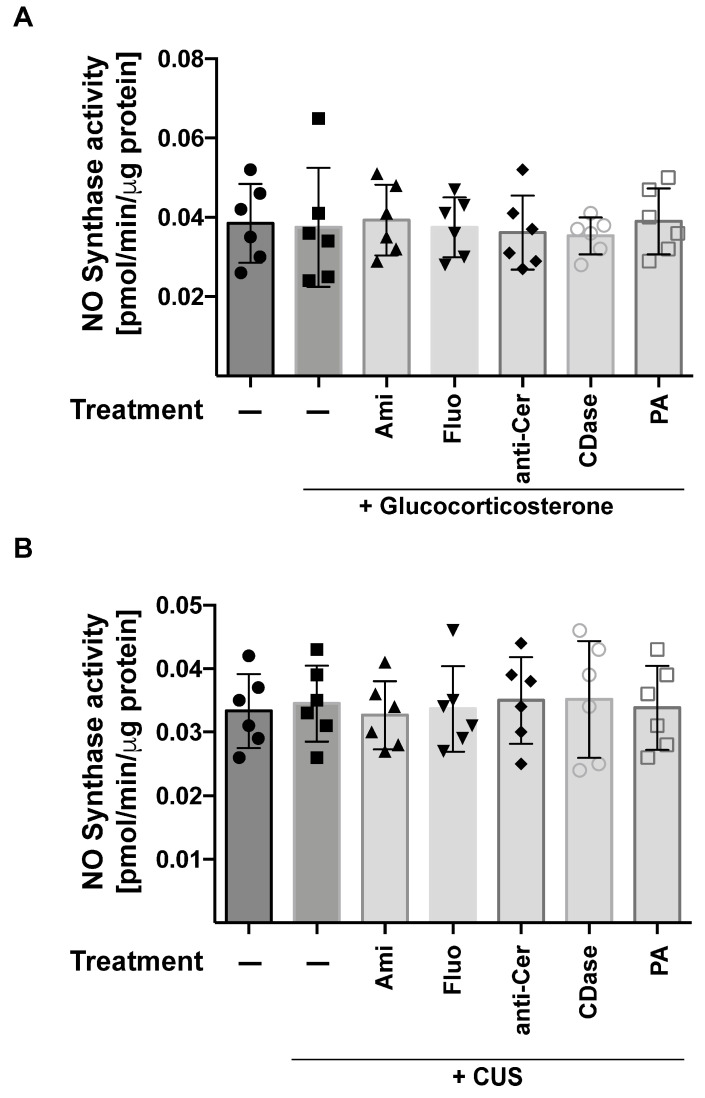

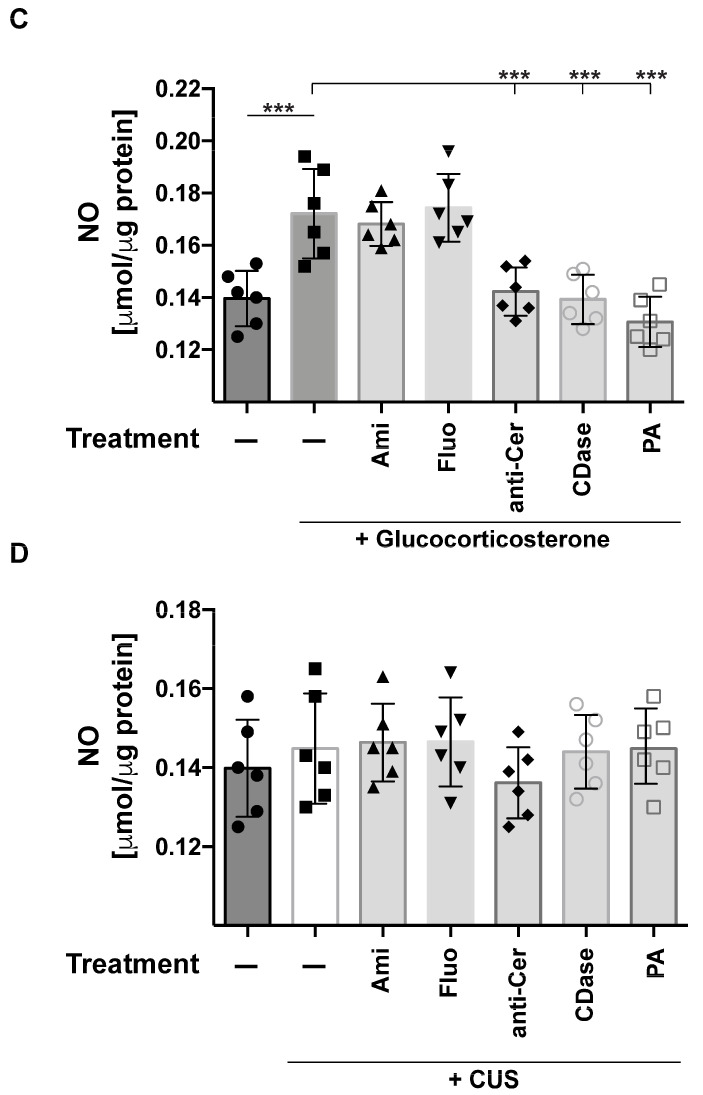
Stress does not induce NO synthase activity. The activity of NO-synthase (**A**,**B**) and the formation of NO (**C**,**D**) in the hippocampus were determined through enzyme activity measurements and a modified Griess reaction, respectively. Mice were stressed using glucocorticosterone (**A**,**C**) or chronic unpredictable stress (CUS) (**B**,**D**) and treated with amitriptyline (Ami), fluoxetine (Fluo), anti-ceramide antibodies (anti-Cer), recombinant ceramidase (CDase), or phosphatidic acid (PA) or were left untreated (−). Shown are the mean ± SD from 6 independent studies; statistical differences were determined using ANOVA with Tukey’s post hoc test; *** *p* < 0.001.

The nitrosylation of proteins requires NO and oxygen radicals. Thus, we tested whether stress induces an activation of NO synthase. The data show that neither glucocorticoid stress nor chronic unpredictable stress or any of the treatments had a significant impact on NO synthase (Figure 3A,B). However, glucocorticoid stress, but not chronic unpredictable stress, induced some limited increase in NO in the hippocampus, which was prevented by treatment with anti-ceramide antibodies, ceramidase, or phosphatidic acid (Figure 3C,D).

### 2.3. Stress-Induced Tyrosine Nitrosylation Is Mediated by an Increased Activity of Peroxidases and Catalase

**Figure 4:**
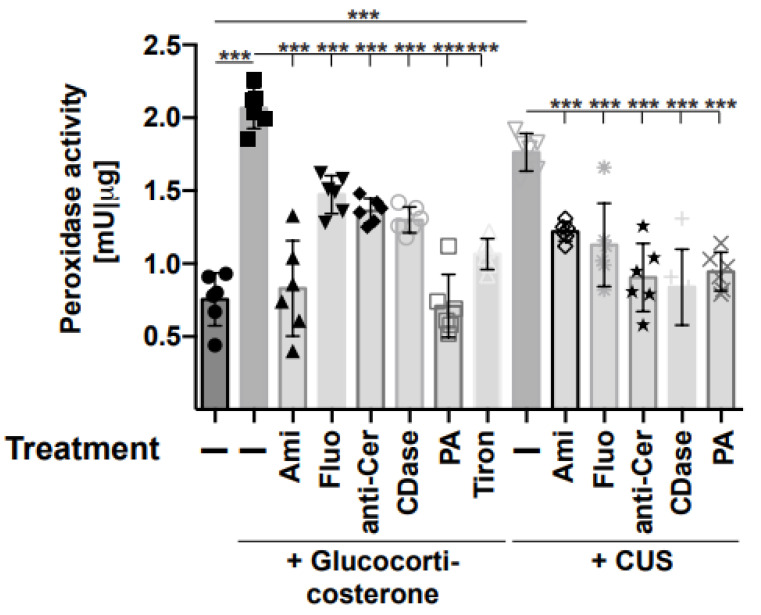
Stress induces peroxidases, which is prevented by anti-depressive treatments. Mice were stressed using glucocorticosterone or chronic unpredictable stress (CUS) and treated with amitriptyline (Ami), fluoxetine (Fluo), anti-ceramide antibodies (anti-Cer), recombinant ceramidase (CDase), phosphatidic acid (PA), or Tiron or were left untreated (−). Mice that were not stressed and left untreated served as controls. The activity of peroxidase was determined in homogenates of the hippocampus using a commercial assay kit. Given are the mean ± SD from 6 independent studies; statistical differences were determined using ANOVA with Tukey’s post hoc test; *** *p* < 0.001.

**Figure 5:**
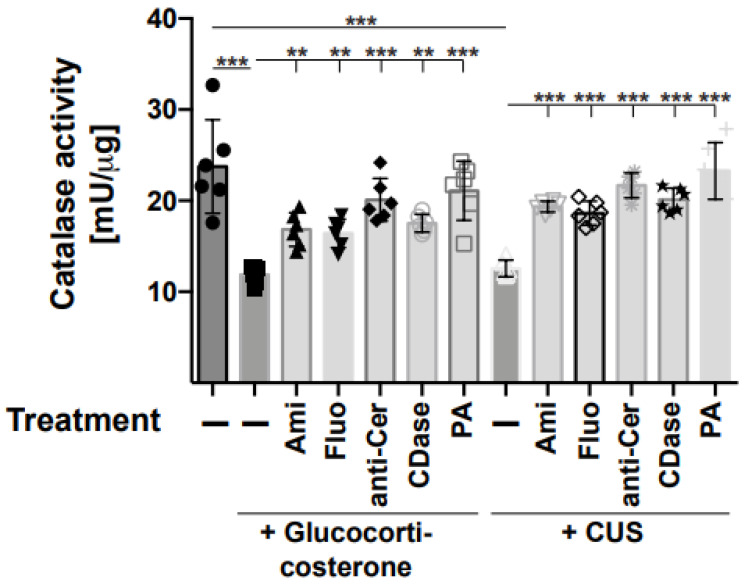
Stress inhibits catalase in the hippocampus, which is prevented through anti-depressive treatments. Catalase activity was determined in hippocampus extracts obtained from mice that were stressed using glucocorticosterone or chronic unpredictable stress (CUS) and treated with amitriptyline (Ami), fluoxetine (Fluo), anti-ceramide antibodies (anti-Cer), recombinant ceramidase (CDase), or phosphatidic acid (PA) or were left untreated (−). Control mice were not treated at all. Displayed are the mean ± SD from 6 independent studies; statistical differences were determined using ANOVA with Tukey’s post hoc test; ** *p* < 0.01, *** *p* < 0.001.

Thus, we focused on the formation of oxygen radicals, which can accumulate in a cell or tissue either through increased formation, reduced consumption, or a combination of both. Therefore, we determined the activity of peroxidase, producing oxygen radicals, as well as the activity of catalase-degrading oxygen radicals in the hippocampus of stressed and unstressed mice. The results demonstrate that stress induced an activation of peroxidase in the hippocampus (Figure 4), which was prevented through treatment of the mice with amitriptyline, fluoxetine, or injections of anti-ceramide antibodies, recombinant ceramidase, or phosphatidic acid (Figure 4). Treatment with the anti-oxidant Tiron also reduced peroxidase activity and served as a positive control (Figure 4). The activity of catalase was inhibited by stress and also corrected through treatment with amitriptyline, fluoxetine, or injections of anti-ceramide antibodies, recombinant ceramidase, or phosphatidic acid (Figure 5).

### 2.4. Inhibition of Stress-Induced Tyrosine Nitrosylation Improves Symptoms of Experimental MDD

**Figure 6:**
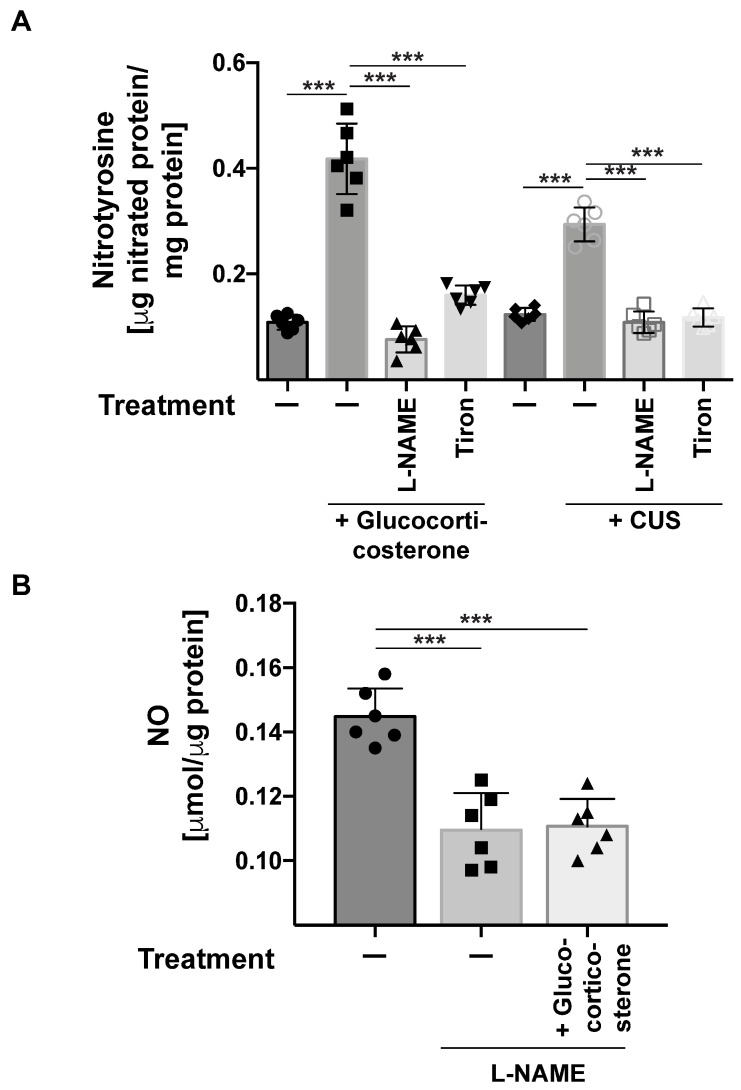
Inhibitors of NO-synthases or oxygen radicals prevent stress-induced tyrosine nitrosylation. (**A**) Inhibition of NO-synthases with L-NAME or peroxidases with Tiron prevents tyrosine nitrosylation in the hippocampus of mice stressed using gluococorticosterone or chronic unpredictable stress (CUS). (**B**) Controls show that L-NAME prevented the formation of NO in the hippocampus. NO was determined in hippocampus extracts from non-stressed mice or mice stressed using gluocorticosterone and treated with L-NAME. In panels A and B, we show mean ± SD from 6 independent studies; statistical differences were determined using ANOVA with Tukey’s post hoc test; *** *p* < 0.001.

**Figure 7:**
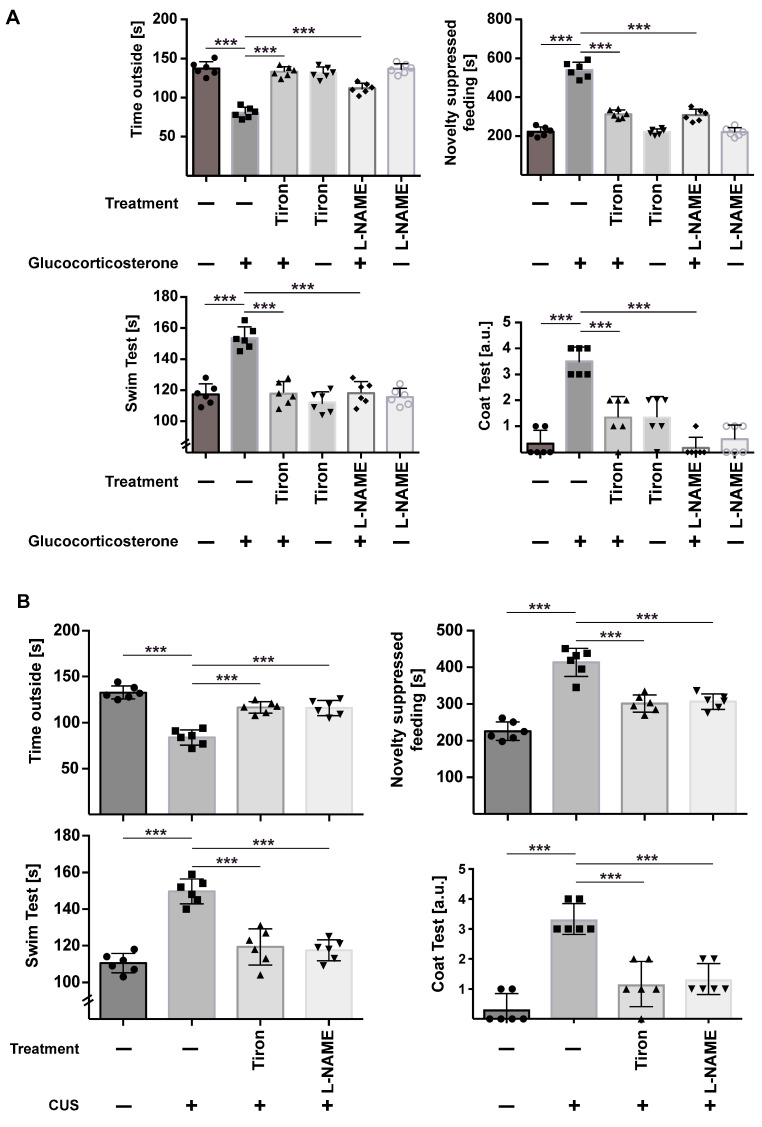
Inhibitors of NO-synthases or oxygen radicals revert stress-induced depressive behavior. Mice were stressed using glucocorticosterone (**A**) or chronic unpredictable stress (CUS) (**B**) and treated with Tiron or L-NAME. Mice that were not stressed and treated with Tiron or L-NAME or left completely untreated served as controls. We then determined the behavior of the mice in the light–dark test, the novelty-suppressed feeding test, the water-swim test, and the coat test. Given are the mean ± SD from 6 independent studies; statistical differences were determined using ANOVA with Tukey’s post hoc test; *** *p* < 0.001.

To test the significance of nitrosylation of proteins in stress-induced major depression, we treated stressed mice with the inhibitor of NO-Synthase L-NAME and the anti-oxidant Tiron and determined the nitrotyrosine concentration in the hippocampus and the behavior of the mice. L-NAME and Tiron prevented the increase in nitrotyrosine concentrations in the brain after stress (Figure 6) and, most importantly, reverted depressed behavior in stressed mice (Figure 7A,B).

## 3. Discussion

Here, we provide evidence that two different forms of stress, i.e., application of glucocorticoid and chronic unpredictable environmental stress, induce an increased nitro-sylation of proteins in the hippocampus. We further demonstrate that protein nitrosylation in the hippocampus of stressed mice seems to be caused by an increased activity of peroxidases and a reduced activity of catalase, while NO-synthases are not altered. Importantly, nitrosylation is prevented by classical antidepressants such as amitriptyline or fluoxetine, but also the neutralization of blood plasma ceramide upon i.v. injection of anti-ceramide antibodies or recombinant ceramidase, or the application of phosphatidic acid to compensate for the ceramide-mediated inhibition of phospholipase D in the hippocampus of stressed mice. Pharmacological inhibition of nitrosylation prevented nitrosylation in the hippocampus and reverted the depressed behavior of stressed mice.

While oxidative stress has been implied to be important in the pathomechanism of MDD [33,34], much less is known about the potential role of the nitrosylation of proteins in the hippocampus. Several studies have previously shown that autophagy is reduced in MDD and this impairment of autophagy is corrected by treatment with antidepressants [35,36]. It is possible that the accumulation of nitrosylated proteins in the hippocampus is a consequence of reduced autophagy, resulting in an accumulation of altered proteins. However, the observation that stress induces an activation of peroxidase and an inhibition of catalase activities suggests that the formation of nitrotyrosine residues is not simply a consequence of altered autophagy, but rather an active process induced by stress.

We have previously shown that stress triggers an increase in ceramide-containing exosomes in the blood plasma, resulting in an inhibition of phospholipase D and reduced formation of phosphatidic acid in the hippocampus [26]. Phosphatidic acid has been previously shown to inhibit the activity of peroxidases [37]. Since we demonstrate that treatment with phosphatidic acid reduces the formation of nitrotyrosine residues in the hippocampus, we assume that the decrease in phosphatidic acid in the hippocampus of stressed mice results in a dis-inhibition/activation of peroxidases, resulting in increased concentrations of oxygen radicals and therefore the nitrosylation of proteins. However, it is certainly possible that the combination of reduced autophagy or modified/dysfunctional proteins through autophagy and the activation of peroxidases determine the levels of nitrosylation in MDD.

Studies in *C. elegans* previously revealed that PLD, which releases phosphatidic acid, regulates the expression of catalase [38]. It was shown that a decrease in PLD with the presumable consequence of the reduced formation of phosphatidic acid resulted in decreased expression of catalase [38]. Thus, the ceramide-mediated inhibition of PLD in stressed mice [26] might be a possible explanation for the reduced activity of catalase in the hippocampus, which is then corrected through treatment with phosphatidic acid.

It is interesting to note that all treatments of MDD, i.e., treatment with classical antidepressants such as amitriptyline and fluoxetine, but also treatments to neutralize ceramide in the blood plasma such as injections of anti-ceramide antibodies or recombinant ceramidase, or the application of phosphatidic acid to restore its concentrations in the hippocampus, prevented the nitrosylation of proteins in the hippocampus. This may indicate that nitrosylation is a common pathway of major depression that is targeted by different modes of treatment, i.e., drugs that neutralize or compensate ceramide or classical antidepressants. It might also be possible that nitrosylation is a relatively downstream event in MDD and therefore several treatments finally correct this alteration. We assume that phosphatidic acid acts directly on peroxidases. Amitriptyline and fluoxetine have not been reported to alter the activity of peroxidases, and we assume that the drugs alter different cellular mechanisms to correct nitrosylation in the hippocampus. In this context, it is interesting to note that antidepressants promote cellular autophagy via inhibition of the acid sphingomyelinase and activation of protein phosphatase 2A [25]. Thus, these drugs might reduce nitrosylation through an increased degradation of modified proteins even if the increased activity of peroxidases persists.

We demonstrate that the treatment of mice with the NO-synthase-inhibitor L-NAME [39] and the anti-oxidant Tiron [40] corrects the stress-induced behavior and increases nitrosylation in the hippocampus. Although L-NAME is a relatively specific inhibitor of NO synthases and therefore affects the formation of NO-radicals, it certainly is a pleiotropic drug, and other effects of the drug, for instance, on endothelial cells, may contribute to the reduction in nitrosylation and the improved behavior of stressed mice. Likewise, the anti-oxidant Tiron does prevent the effects of oxygen radicals, but it is a nonspecific drug. Nevertheless, both drugs, although targeting different pathways, have very similar effects on the nitrosylation of proteins in the hippocampus and on the behavior of stressed mice, possibly arguing that nitrosylation is important for MDD. Unfortunately, no specific drug targeting nitrated proteins in the brain is presently available and, therefore, further studies that identify specific proteins altered by nitrosylation and their role in MDD are required to unambiguously prove this hypothesis.

In addition to the oxidation and nitrosylation of proteins, it might be possible that lipids are also oxidized in stress-induced MDD. This was not investigated in the present manuscript and requires separate studies that are beyond the present study. However, the inhibition of NO formation and peroxidase would also prevent or at least reduce lipid oxidation, and this effect might contribute to the observed inhibition of depressed behavior by Tiron and L-NAME.

It is interesting to note that a variety of molecular mechanisms have been implicated in the pathogenesis of major depression, as described above. It is difficult to explain how a specific alteration affects that many molecular mechanisms, while the induction of tyrosine nitrosylation might simultaneously affect many molecules in neurons and/or glia cells of the hippocampus and therefore alter several aspects of neuronal and glial physiology. Therefore, the present data might be an explanation for a variety of biochemical and molecular changes in the hippocampus of depressed mice.

## 4. Material and Methods

### 4.1. Overview of Used Methods

Measurements of tyrosine nitrosylation in protein of the hippocampus were carried out using the following:

1. ELISA from Hycult (#HK501)

2. ELISA from AFG Bioscience (#EK700342)

3. Confocal microscopy

The determination of NO in the hippocampus was carried out to test whether tyrosine nitrosylation in stressed mice is caused by increased NO.

Nitric oxide assay kit was from Abcam, Cambridge, UK (#ab272517)

Enzyme assays in the hippocampus were carried out to determine mechanisms of tyrosine nitrosylation:

1. Nitric-oxide-synthase activity: enzyme activity kit from Abcam (#ab211085)

2. Peroxidase activity assay: enzyme activity kit from Abcam (#ab155895)

3. Catalase activity assay: enzyme activity kit from Invitrogen (#EIACATC)

Behavioral studies were carried out to determine the functional role of tyrosine nitrosylation for MDD.

### 4.2. Mice and Treatments

All animal studies were performed with permission from the State Agency for Nature, Environment and Consumer Protection (LANUV) NRW, Recklinghausen, Germany (#81-02.04.2017.A084, #81-02.04.2019.A211, #81-02.04.2018.A413, and 81-02.2019.A003) and the local IACUC, Medical College, University of Cincinnati, USA.

We used C57BL/6 wildtype mice with an age of 8–12 weeks and weight between 25 and 30 g. Mice were housed in isolated ventilated cages in an enriched environment and had free access to food and water. Mice were bred at the animal facilities of the University of Duisburg-Essen, University Hospital, and the Medical College of the University of Cincinnati. Mice were treated with glucocorticosterone (Sigma, Deisenhofen, Germany) administered via the drinking water at 100 mg/L for 6 days. Mice were exposed to chronic unpredictable stress for 15 days, i.e., the mice were challenged for 15 days with a reversal of the light/dark cycle, 3 h of 45° tilting of the cage twice each week, shaking at 125 rpm for 45 min, food deprivation for 14 h, predator sounds for 15 min, or wet cages for 1 h with two forms of stress per day in a randomized (unpredictable) order. We used 6 mice per group. No animals were excluded. Mice were sacrificed through cervical dislocation.

All experiments were performed according to the FELASA regulations and we also followed the ARRIVE guidelines. Mice were scored daily for their health status.

Amitriptyline (Sigma) and fluoxetine (Sigma) were applied in the drinking water at 120 mg/L for 11 days prior to any studies.

All other drugs were intravenously injected 24 h and 12 h prior to any further analysis. Anti-ceramide antibodies clone S58-9 (purified IgM anti-ceramide antibodies, Glycobiotech, Kuekel, Germany, cat. no. #MAB0011) were intravenously injected at 0.08 μg/g body weight.

Recombinant neutral ceramidase (Asah2, R&D, 3000 pmol/min/μg, cat. no. #3557-AH) was i.v. injected at 40 ng/g body weight.

Phosphatidic acid (Sigma, cat. no. #1535722) was suspended at 1 mg/mL in PBS, sonicated for 10 min in a bath sonicator to obtain a stable suspension, and i.v. injected at 4 μg/g body weight.

L-NAME (Sigma) was intraperitoneally (i.p.) injected at a dose of 4 mg/kg. We injected L-NAME 24 and 12 h prior to analysis.

Tiron (Fluka Chemie) was injected i.p. at a dose of 100 mg/kg, 24 and 12 h prior to analysis of the hippocampus.

### 4.3. ELISA for Nitrotyrosine/Nitrated Protein

We employed two independent ELISAs to detect nitrotyrosine in the hippocampus. The first ELISA was obtained from Hycult (#HK501) and the second ELISA was obtained from AFG Bioscience (#EK700342). Resting or stressed mice were treated with amitriptyline, fluoxetine, anti-ceramide antibodies, recombinant ceramidase, phosphatidic acid, L-NAME, or Tiron, or were left untreated. They were sacrificed through cervical dislocation; the brain was removed; and the hippocampus was prepared, removed, and immediately shock-frozen in liquid nitrogen. The tissue was homogenized using a tip sonicator for 10 s in 300 μL of 0.9% NaCl. Sonication was repeated twice. Samples were then immediately used to quantify nitrotyrosine. The assays use different anti-nitrotyrosine antibodies to detect nitrotyrosine residues in the lysates. We exactly followed the instructions of the vendor to perform the ELISA. ELISA samples were analyzed on a FLUOstar Omega ELISA plate reader.

### 4.4. Protein Measurements

We quantified protein concentrations in the samples by using the BioRad Protein Assay Dye (cat. no. #500006). Data served to normalize ELISA studies.

### 4.5. Confocal Microscopy

Resting or stressed mice were treated as indicated or left untreated, euthanized, and perfused via the left heart for 2 min with 0.9% NaCl. Tissues were then fixed through immediate perfusion with 4% paraformaldehyde (PFA) buffered in PBS (pH 7.3) for 15 min. Brains were then carefully removed, split along the great fissure, and fixed for an additional 36 h in 4% buffered PFA in phosphate-buffered saline (PBS). Tissues were embedded in paraffin and serially sectioned at 6 μM. The sections were then dewaxed in Xylol and an ethanol series, washed in water, and incubated for 30 min with pepsin (Digest All, Invitrogen, Darmstadt, Germany) at 37 °C to recover antigens. Samples were washed in PBS, and unspecific binding sites were blocked for 10 min with PBS and 5% fetal calf serum (FCS). The samples were washed once in HEPES/Saline (H/S; 132 mM NaCl, 20 mM HEPES (pH 7.4), 5 mM KCl, 1 mM CaCl_2_, 0.7 mM MgCl_2_, 0.8 mM MgSO_4_) and then incubated for 45 min at room temperature with mouse monoclonal anti-nitrotyrosine antibodies (1:200, clone 306-507; R&D). The anti-nitrotyrosine antibodies were diluted 1:250 in H/S, supplemented with 1% FCS. The samples were washed 3 times for 5 min each in PBS containing 0.05% Tween 20, once in PBS, and then stained for 45 min at room temperature with Cy3-coupled donkey anti-mouse IgG F(ab)_2_ fragments (diluted 1:500; Jackson ImmunoResearch, cat. No. #715-166-150). Samples were finally washed again 3 times in PBS supplemented with 0.05% Tween 20 and once in PBS, embedded in Mowiol, and analyzed on a LEICA TCS SL confocal microscope. Control stainings with isotype-matched control antibodies showed very weak or no signals and served as specificity controls.

The fluorescence was quantified using Photoshop. The investigator was blinded to the identity of the samples.

### 4.6. NO-Measurements

NO was determined using a nitric oxide assay kit from Abcam (#ab272517). This assay consists of a modified and improved Griess method and determines NO production by measuring the reduction of nitrate to nitrite. The detection minimum is 600 nM. We exactly followed the protocol provided by the vendor. Samples were analyzed on a FLUOstar Omega Plate reader.

### 4.7. Measurement of Nitric-Oxide-Synthase Activity

We employed a commercial kit (Abcam, #211085) that determines the activity of intracellular NO synthase. It is a non-ratiometric method to determine enzyme activity. The kit uses a fluorescent dye to detect newly formed NO to produce fluorescence with an excitation wavelength of 485 nm and emission wavelength of 530 nm. Fluorescence was quantified on a FLUOstar Omega.

### 4.8. Peroxidase Activity Assay

The peroxidase activity assay employs an OxiRed probe that reacts with peroxide (H_2_O_2_) in a 1:1 stochiometric ratio to generate the oxidation product resorufin. The amount of generated resorufin was quantified using colorimetry on a FLUOstar Omega using excitation at 570 nm. We exactly followed the instructions of the vendor (Abcam, #ab155895).

### 4.9. Catalase

To determine catalase activity, we used a commercial kit from Invitrogen (#EIACATC). In this assay, hydrogen peroxide horseradish peroxidase converts a colorless substrate to a colored product, if hydrogen peroxide is present. Analysis of the samples at 560 nm and comparison with a standard curve using a bovine catalase standard serves to quantify hydrogen peroxide in the samples. We exactly followed the protocol provided by the vendor and used a FLUOstar Omega for analysis.

### 4.10. Behavioral Studies

Behavioral tests were performed under standardized conditions as previously described. All experiments were performed between 6.30 a.m. and 9 a.m., under diffuse indirect room light. In the novelty-suppressed feeding test, also named latency-to-feed test, experimental mice were fasted for 24 h, placed in a new arena with one piece of food on a white piece of paper in the middle of the arena, and the time during which the mice explored the new environment before they began eating was recorded. In the light/dark box test, test mice were placed in a dark and safe compartment that was connected via a 5 cm × 5 cm rounded-corner aperture to an open area, which was illuminated and, thus, aversive for the mice. The result of the test is expressed as the time that the mouse spent in the open arena during a 5 min observation period. In a similar test, the open-field arena test, experimental mice were placed near to the wall of a new arena of 50 cm × 50 cm × 30 cm (height) dimensions, and the time during a 30 min observation the animal was more than 10 cm away from the wall was recorded. The appearance of the coat, i.e., groomed vs. unkempt coat, on the head, neck, back, and ventrum of the mice was scored with either a zero for normal status or a 1 for unkempt status in the coat state test. Finally, we also performed the forced swim test, in which mice are placed in a cylinder filled with water (21–23 °C) for 15 min as the 1st step. The test is then performed 24 h later: The mice are again placed in a water-filled cylinder for 6 min. The time of immobility during the last 4 min of the test is then recorded and given as result.

### 4.11. Quantification and Statistical Analysis

Data are expressed as arithmetic means ± SD. One-way ANOVA followed by post hoc Tukey test for all pairwise comparisons was employed to compare continuous variables from independent groups with one variable (treatment). The *p* values for the pairwise comparisons were calculated after Bonferroni correction for multiple testing. All values were tested for normal distribution using the Kolmogorov–Smirnov test. Statistical significance was set at a *p* value of 0.05 or lower (two-tailed). Outliers were not removed. To reduce the number of mice to the required minimum, we performed extensive sample size planning. This was based on two-sided Wilcoxon–Mann–Whitney tests using the free version of G*Power, Version 3.1.7, University of Duesseldorf, Germany. The alpha error was set at 0.05, the power at 0.95, and the effect size between 0.5 and 0.9, depending on the assay type. Investigators were blinded to the results of histologic analyses and to animal identity. We performed two simple randomizations: Before the experiments, animals were randomly assigned to cages by a technician who was not involved in the experiments; thus, the mice were purely randomly assigned for every experiment. Cages were then randomly assigned to the various experimental groups.

## 5. Conclusions

The present study provides evidence that stress induces tyrosine nitrosylation in the hippocampus. Tyrosine nitrosylation is mediated by increased peroxidase and decreased catalase activity in the hippocampus upon stress. The antioxidant Tiron and the NO-synthase inhibitor L-NAME prevent stress-induced tyrosine nitrosylation and improve depressed behavior in experimental MDD. However, this study has three major limitations: Tyrosine nitrosylation very likely occurs in many proteins, and individual target proteins remain to be identified. Second, lipids might also be altered upon increased peroxidase activity and the role of lipid modifications in MDD also remains to be defined. Finally, the inhibitors Tiron and L-NAME are not specific and a potential role of tyrosine nitrosylation in depressed behavior needs to be confirmed with more specific reagents.

## Data Availability

All data are provided in the manuscript.
